# Magma fragmentation and particle size distributions in low intensity mafic explosions: the July/August 2015 Piton de la Fournaise eruption

**DOI:** 10.1038/s41598-020-69976-y

**Published:** 2020-08-18

**Authors:** Matthew J. Edwards, Laura Pioli, Andrew J. L. Harris, Lucia Gurioli, Simon Thivet

**Affiliations:** 1 Département des Sciences de la Terre, Université de Genève, Geneva, Switzerland; 2 Dipartimento di Scienze Chimiche e Geologiche, Università Degli Studi di Cagliari, Cagliari, Italy; 3 Laboratoire Magmas et Volcans, Université Clermont Auvergne, CNRS, OPGC, Clermont-Ferrand, France

**Keywords:** Volcanology, Natural hazards

## Abstract

Understanding magma fragmentation mechanisms in explosive eruptions is a key requirement for volcanic hazard assessment, eruption management and risk mitigation. This paper focuses on a type case small explosivity eruption (July–August 2015 eruption of Piton de la Fournaise). These eruptions, despite being often overlooked, are exceedingly frequent on local-to-global scales and constitute a significant hazard in vent-proximal areas, which are often populated by guides, tourists and, indeed, volcanologists due to their accessibility. The explosions presented here are ideal cases for the study of the dynamics of magma fragmentation and how it relates to the size distribution of scoria generated at the vent. We documented these events visually and thermally, and characterised the products through sample-return. This allowed us to describe small-scale gas bursts sending ejecta up to 30 m during intermittent lava fountains. Surface tension instabilities and inertial forces played a major role in fragmentation processes and generated particles with coarse-skewed distributions and median diameters ranging from − 8 to − 10 ϕ. However, with time distributions of particles in the most energetic fountains shifted towards more symmetrical shapes as median grains sizes became finer. Analyses of sequences of images demonstrate that the evolution of particle size distributions with time is due to instability of magma droplets and (in-flight) fragmentation.

## Introduction

Mafic explosive volcanism is traditionally overlooked with respect to more energetic, higher intensity and destructive silicic volcanism. Several events in the last years (Kilauea^1^, Mount Etna^2^ have however demonstrated that significant hazard is associated with such low intensity basaltic eruptions^3,4^ and the dynamics and impact of mafic explosive events has been the subject of several studies^5–11^. These studies have highlighted that accurate assessment of the hazard associated with mafic explosive activity requires greater understanding of the volcanic events from precursory activity to fragmentation and pyroclast accumulation and sedimentation.

Mafic magma fragmentation shows a large variability, ranging from poorly to highly efficient. Poorly efficient fragmentation in low intensity eruptions (i.e. those not driven by significant gas overpressure) relates to the formation of large bomb-sized fragments (smaller median phi) which fall around the vent, and, if accumulation rates are high (and clast cooling rates low) are capable of coalescing into spatter-fed lava flows which collect most of the fragmented magma^12–14^. More efficient fragmentation, typical of the moderate intensity Strombolian and violent Strombolian explosions (or transitional regimes^15–17^ produces lapilli to ash-sized fragments which either form scoria cones, or plumes which settle forming tephra-fall deposits or disperse in the atmosphere^7,18,19^. In the high intensity Plinian and Subplinian eruptions, fragmentation is highly efficient resulting in total grain size distributions (TGSD) that are similar to those of other more felsic magmas^20–22^ (i.e. the median diameters of the distributions are lapilli-sized). At the moment, magma fragmentation mechanisms and efficiency in low viscosity, mafic systems are poorly quantified, although the role of magma vesicularity (in terms of bubble size distribution^22^) and crystallinity (because it increases bulk magma viscosity^23^) has been demonstrated. Magma viscosity, diameter, density, surface tension and velocity are recognised as relevant parameters in magma fragmentation. Fragmentation of low viscosity magmas is controlled by surface tension, viscous and shearing forces; among the main dimensionless parameters used to constrain the dynamics of the breakup regimes are the Reynolds and Ohnesorge numbers^21,24,25^ which are quantifying the relative role of these forces^26–28^; however, very few data are available from real eruptions^29^.

Although small-scale activity has been documented by several geophysical and textural studies^8,30–35^ little is known about the specific dynamics of all these fragmentation regimes, specifically what controls their stability and their transitions. For this reason, it is not possible to relate specific particle size distributions to eruption dynamics, and the size distributions of particles generated at the vent are themselves considered as eruption source parameters^36,37^ (i.e. they cannot be estimated for given eruption conditions). Total grain size distributions are instead commonly reconstructed from tephra deposits through statistical methods, e.g., by weighted-average of sample GSDs, arbitrary sectorisation, or by Voronoi tessellation^38,39^. These methods rely on the ability to sample a deposit, an often difficult to impossible procedure in small-scale eruptions where deposits are ephemeral and/or rapidly covered by subsequent lava or scoria, and thus rarely documented^40,41^. For this reason, a more efficient assessment of GSDs can be done at the vent by video and thermal monitoring^29,42,43^.

**Figure 1:**
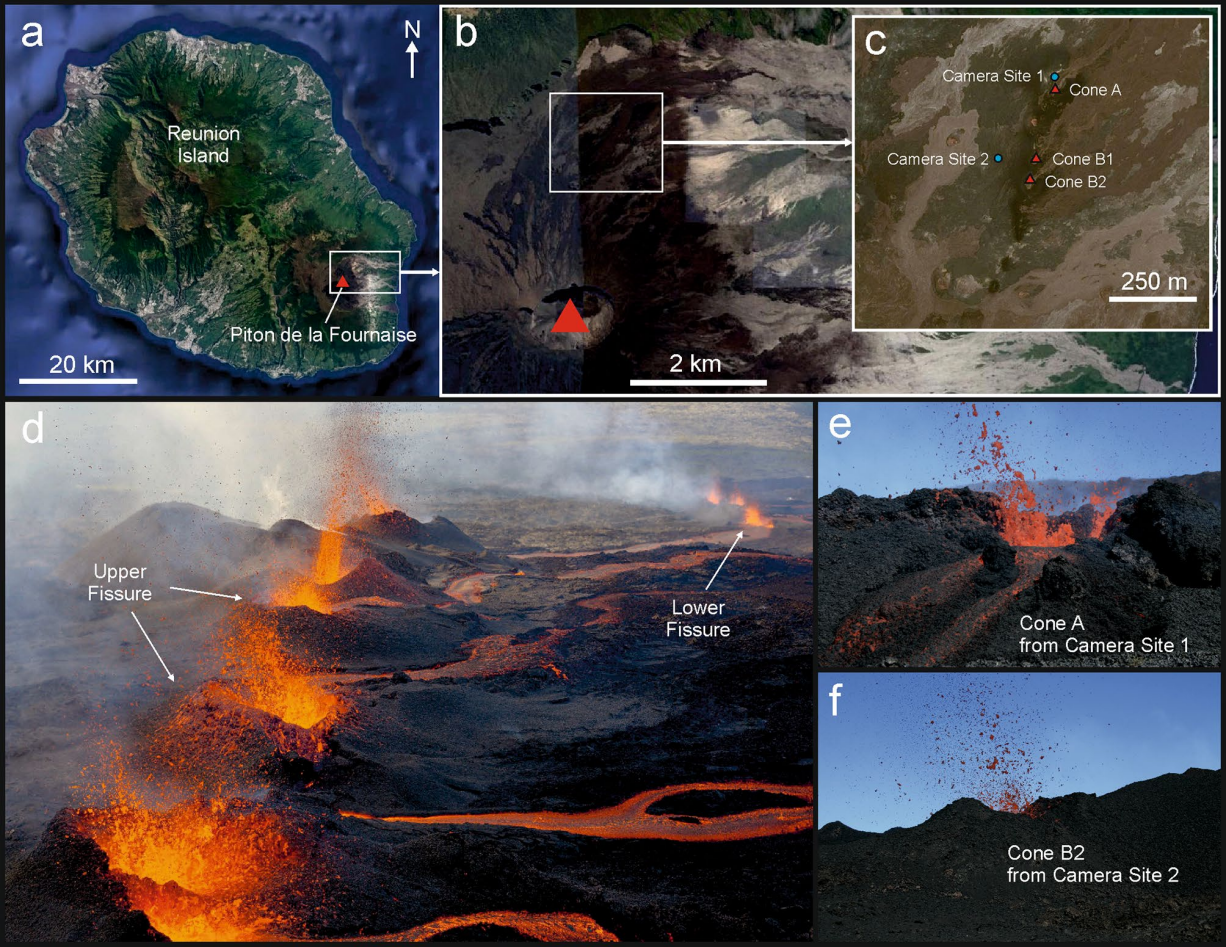
Overview of PdF volcano and activity of July–August 2015. (**a**) Location of volcano on Reunion Island with inset (**b**) showing caldera position and inset (**c**) the location of activity with positions of active cones of the upper fissure and recording positions. Photographs of activity on 31 July (**d**) and the views of the cones on 1 August (**e** and **f**). Map data for (**a**), (**b**) and (**c**): Google, Maxar Technologies Photo (**d**) taken by Serge Gèlabert.

**Figure 2:**
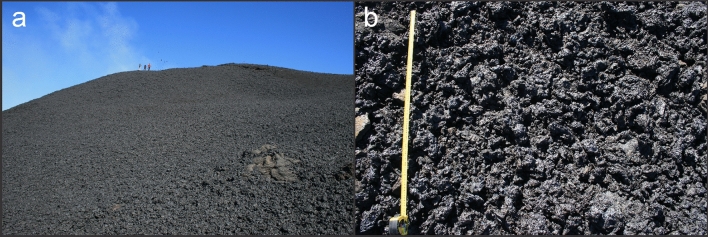
Deposit images of the PdF eruption of July–August 2015. Broad view of the deposit looking towards Camera Site 1 with location of monitoring activities (researchers on the cone rim) and Cone A (**a**) and a closeup of the deposit shown with 1 m tape measure (**b**).

In this paper, we analyse magmatic fragmentation in discrete basaltic explosions by studying the July–August 2015 eruption of Piton de la Fournaise (PdF) volcano. This eruption consisted of multiple vents which produced lava fountaining, discrete explosions and lava flows. The eruption began at 09:20 (all times are local, GMT + 4) on 31 July 2015 from two en echelon eruptive fissures where low level (20–30 m high) fountaining fed lava flows at rates of 22 ± 8 m^3^/s (Fig. 1)^44^. By the morning of 1 August activity had become localized to several vents distributed along the upper fissure and eruption rate dropped to 0.8 ± 0.4 m^3^/s (Fig. 1)^44^. By the afternoon, activity was limited to the lowermost three cones of the Southern fissure (A, B-I and B-II, Fig. 1). Cones A and B-II are those targeted here. Cone A, at the lowest end of the fissure, was large enough to show the free magma surface at the ground level and fed a lava channel that exited the cone to the NE. The magma surface was disrupted by discrete explosions every few seconds which ejected lapilli and bombs to a height and distance of up to a few tens of meters. Cones B-I and B-II were located ~ 100 m higher up on the fissure feeding Cone A and were active with explosions of the same character as Cone A. Activity waned though the following night, with the eruption ending around 10:30 during the morning of 2 August. Careful description and parameterisation of this style of activity using visible and thermal images was obtained due to a clear line of sight of the vents from distances of ~ 50–120 m, which allowed for observation of the free magma surface and its fragmentation, a view which is often obscured by conduit/vent/cone geometry. We use this dataset in parallel with tephra collected during the eruption (Fig. 2) to investigate the evolution of particle size distributions across individual explosions and highlight the mechanisms controlling fragmentation of the magma jet into scoria particles. We detail our discussion on the two end-member explosion types observed by video and thermal monitoring.

## Results

### Eruption parameters and magma properties

Channel width of the lava flow fed from the active vent considered here was 2.7 ± 0.1 m, corresponding to a cross-sectional area of 3.75 m^2^ (assuming a rectangular channel, with depth of half of the channel width). Maximum lava flow velocity was calculated as 0.17 ± 0.05 m/s by tracking surface clasts in the centre of the lava channel. Overall this equates to a lava effusion rate of 0.64 ± 0.20 m^3^/s. The vent diameter was 4.5 ± 0.5 m equating to an area of about 15.9 ± 3.7 m^2^ (based on the observed circularity of the vent). Visual observation of the eruption dynamics means that we can consider the lava emission rate as equal to the volume flow rate at the vent (as most fragmented magma fell back into the vent and eventually contributed to the lava flow). Given a measured density of 1,320 ± 40 kg/m^3^ for the corresponding water-quenched lava flow sample^35,44^ the volume flow rate converts to a mass flow rate of 845 ± 26 kg/s. Magma viscosity was estimated based on the lava and scoria groundmass compositions (which are very similar^45^ and crystallinity (measured on scoria collected on the same day (and during the same eruptive activity) and tephra vesicularity. They also take into account the lava temperature of 1,146 °C measured in the field. The calculations^46–48^ gives a melt viscosity comprised between 27 and 370 Pa s. These parameters equate any given explosion of the PdF eruption on August 1 to a Reynolds number of range 2.3 × 10^1^ to 1.3 × 10^3^ and an Ohnesorge number of range 2.4 × 10^0^ to 4.1 × 10^1^.

### End-member explosion properties

**Figure 3:**
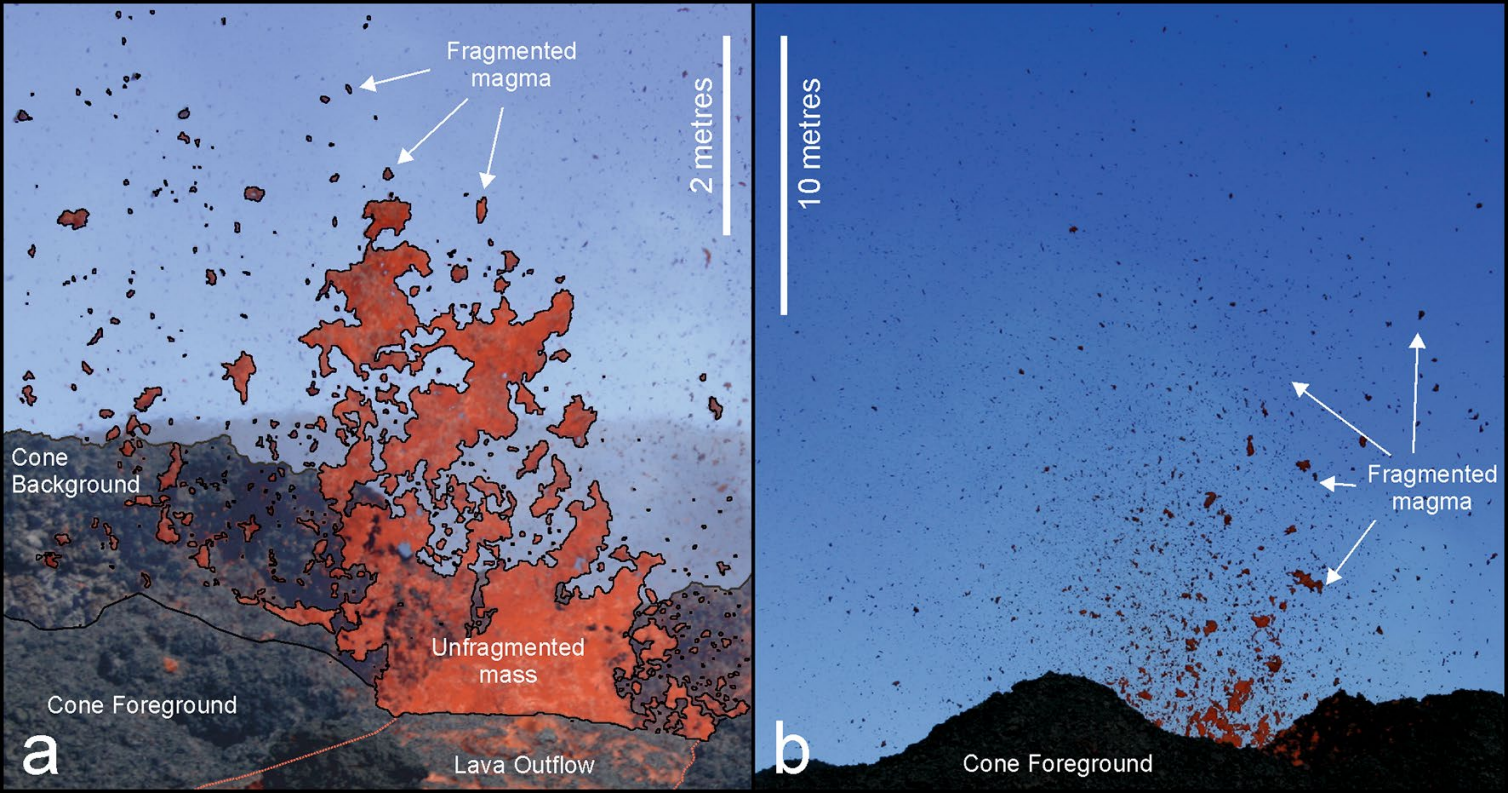
Manifestations of discrete end-member explosions during the waning phase of the July 2015 eruption at PdF. A low intensity explosion is shown in (**a**), and a high intensity explosion is shown in (**b**). Particles are outlined in (**a**) to distinguish them from the background.

The activity observed across the images spans a range of intensities from minor ejections of lava fingers to well-developed explosion fountains reaching 20 m above the vent (Fig. 3). Intensity is scaled by the maximum height reached of the fountain above the vent and dependent on the exit velocity of the magma.

The lowest intensity explosions manifest at the magma surface as an irregularly shaped rising magma mass. The magma fragments as it ascends in the atmosphere and individual particles typically rise to less than 7 m above the vent. The majority of the ejected mass remains connected to the magma surface through incomplete fragmentation and falls back into the vent. The particles, numbering less than 500 and equating for < 0.5 m^3^ of lava, fall laterally to the vent and build up the surrounding cone. The low intensity explosions were analysed at Cone A, where they were best documented due to the geometry of the cone allowing for the visualization of the eruption dynamics directly above the magma surface. Fully documented explosions had durations ranging from 2 to 4 s.

The high intensity explosions manifests at the magma surface as a discrete jet. A jet of fragments develops a few meters above the magma surface, feeding a cloud of particles tens of metres above the vent. In this case, the majority of particles formed are deposited within tens of metres outside of the crater. The complete range of explosion intensities was observed at Cone A during the observation period. These occurred concurrently; the intensity of explosions was not specific to a given timeframe. An even higher intensity explosion is present in only one image at Cone A where, in place of a magma jet, a spray of fragments is visible being ejected directly from the magma surface. This subset was noted but not analysed due to blur in the image caused by the spray velocity relative to the camera shutter speed.

The relatively high intensity activity was analysed at Cone B-II due to the wider field of view of these images. This permitted a more complete capturing of the cloud of fragmented particles above the vent than was possible at Cone A.

**Figure 4:**
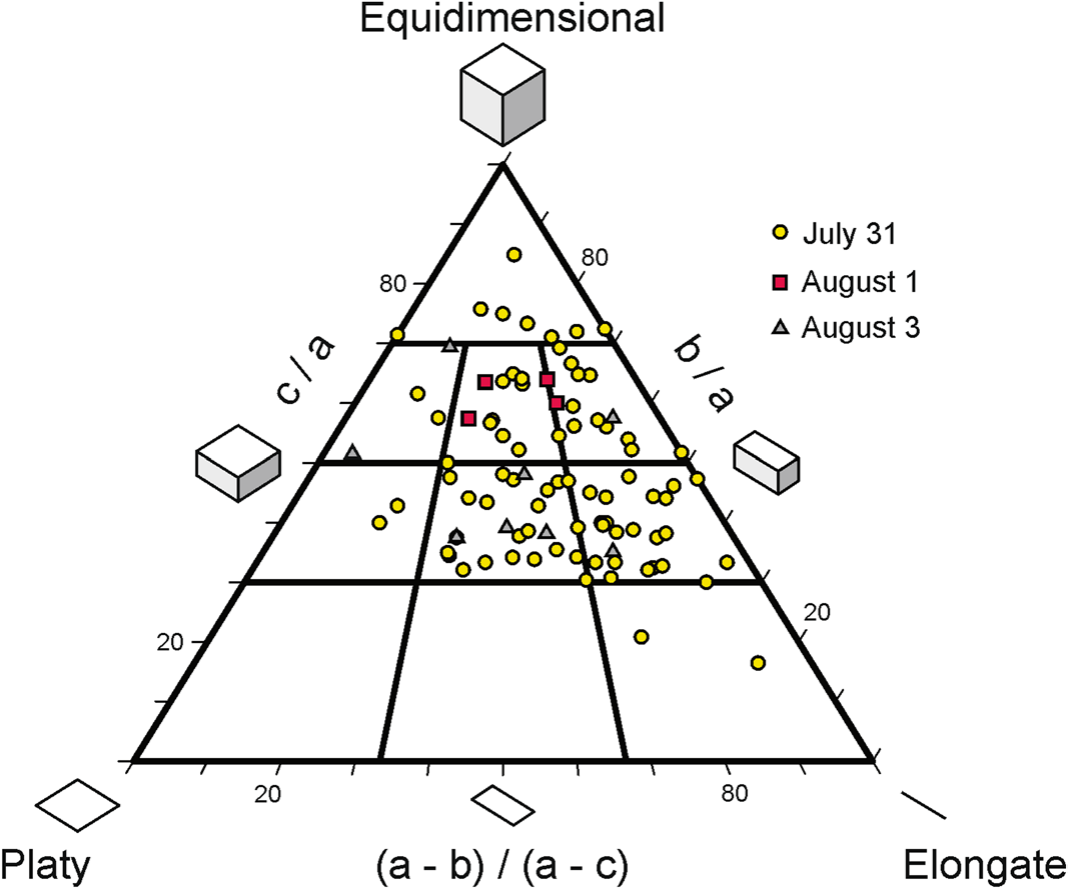
Shape of unbroken clasts collected at the base of the cone on July 31 (yellow circles), August 1 (red squares) and August 3 2015 (grey triangles). Shape triangle from^49^. Letters a, b and c represent the lengths of the three perpendicular axes of clasts.

The shape of the clasts sampled at short distances from the cone in real time (with diameter comprised between − 7 and − 3.5 ϕ) range from equant to bladed and elongated, with all the clasts sampled on August 1 showing a compact bladed shape^49^, i.e. with axes ratios ranging from 0.5 to 0.7. These almost equidimensional to slightly flattened shapes are consistent with the geometry of fragmentation observed at the vent (Fig. 4).

### Explosion fragmentation and efficiency

Both the particle Mdphi and mean phi by mass range between − 8.4 and − 10.1 ϕ, while by the particle median and mean by number show a narrow range between − 5.4 and − 6.3 ϕ (Table 1). The fragmented magma mass varies between 5 × 10^2^ to 5 × 10^4^ kg. The number of particles within an individual explosion is generally between 1 × 10^3^ and 2 × 10^4^, either increasing, oscillating or decreasing in time, suggesting that fragmentation evolves in discrete pulses even within a single event and also that the numbers of particles generated by a single explosion vary by one order of magnitude.

**Table 1:** Parameters for discrete explosions at PdF on 1 August 2015. Fragmented mass is shown for explosions 1 and 2, but is negligible to the total erupted mass for explosions 3–9. Completeness is defined as the fraction of fragmented mass vs total erupted mass.

Cone	Time (GMT 08 Aug 2015)	Explosion number	Image in sequence	Particle Count	Particle max (ϕ)	Size distribution by number			Size distribution by mass			Total erupted mass (kg) with fragmented mass in parentheses	N Particles per m^3^	Completeness
						Median (ϕ)	Mean (ϕ)	Sorting (ϕ)	Mdϕ	Mean (ϕ)	Sorting (ϕ)			
A	8:19:26	1	1	223	− 8.6	− 4.4	− 4.3	1.5	− 8.3	− 8.1	0.7	(1.0 × 10^2^) 1.2 × 10^3^	2,944	0.08
	8:19:27		2	395	− 9.7	− 4.4	− 4.5	1.5	− 9.2	− 9	0.8	(4.5 × 10^2^) 2.4 × 10^3^	1,159	0.19
	8:19:28	2	1	9	− 9.8	− 7.8	− 7.7	1.2	− 9.3	− 9.2	0.7	(1.1 × 10^2^) 1.5 × 10^3^	108	0.07
	8:19:29		2	122	− 9.9	− 6.3	− 6.4	1.1	− 9.4	− 9.3	0.5	(5.2 × 10^2^) 5.4 × 10^3^	310	0.10
	8:19:30		3	245	− 9.4	− 5.8	− 5.7	1.6	− 9.2	− 9.1	0.8	(4.6 × 10^2^) 3.2 × 10^3^	703	0.14
B-II	8:55:16	3	1	891	− 9.6	− 5.9	− 6	0.9	− 8.4	− 8.3	1.2	5.8 × 10^2^	203	~ 1
	8:55:17		2	7,986	− 10.5	− 6	− 6	1	− 9.2	− 9.1	1.5	9.3 × 10^3^	1,133	~ 1
	8:55:18		3	10,075	− 10.8	− 6	− 6	1	− 8.7	− 8.6	1.4	8.7 × 10^3^	1529	~ 1
	9:00:14	4	1	11,005	− 11	− 6.3	− 6.3	1.3	− 9.4	− 9.4	1.3	3.0 × 10^4^	484	~ 1
	9:00:15		2	13,794	− 11.8	− 6.2	− 6.2	1.3	− 10.1	− 9.9	1.5	4.9 × 10^4^	372	~ 1
	9:00:16		3	19,040	− 11.6	− 5.9	− 5.9	1.3	− 10	− 9.7	1.3	4.5 × 10^4^	559	~ 1
	9:05:05	5	1	12,757	− 10.5	− 5.5	− 5.5	1	− 8.9	− 8.7	1.5	4.9 × 10^3^	3,437	~ 1
	9:05:05		2	13,743	− 10.2	− 5.6	− 5.6	1	− 8.3	− 8.3	1.3	5.4 × 10^3^	3,359	~ 1
	9:05:06		3	14,444	− 10.6	− 5.5	− 5.5	1.1	− 8.4	− 8.5	1.5	5.5 × 10^3^	3,467	~ 1
	9:06:51	6	1	5,679	− 11.3	− 6.2	− 6.3	1.1	− 10	− 9.6	1.3	1.4 × 10^4^	535	~ 1
	9:06:51		2	8,609	− 11.6	− 6	− 6.1	1	− 10	− 9.9	1.5	1.9 × 10^4^	598	~ 1
	9:06:51		3	10,890	− 11.3	− 5.7	− 5.8	1.1	− 9.8	− 9.7	1.5	1.7 × 10^4^	846	~ 1
	9:06:52		4	10,478	− 11.4	− 6.1	− 6.2	1	− 9.3	− 9.4	1.6	1.8 × 10^4^	768	~ 1
	9:06:52		5	10,682	− 11.1	− 6	− 6	1	− 9	− 9.1	1.6	1.4 × 10^4^	1,007	~ 1
	9:06:52		6	8,804	− 10.9	− 5.8	− 5.8	1.4	− 8.8	− 8.8	1.3	9.3 × 10^3^	1,250	~ 1
	9:06:35	7	−	6,574	− 10.9	− 5.9	− 6	0.8	− 9.6	− 9.3	1.5	6.7 × 10^3^	1,295	~ 1
	9:06:30	8	−	18,965	− 11.3	− 5.4	− 5.5	1	− 9.1	− 9.2	1.9	9.5 × 10^3^	2,635	~ 1
	9:11:48	9	−	10,598	− 10.6	− 5.8	− 5.8	1.2	− 9	− 8.9	1.4	8.5 × 10^3^	1646	~ 1

The particle median and mean phi by mass for low intensity explosions largely falls within the range for the high intensity explosions at − 8.3 to − 9.4 ϕ. By number these show a greater variation of between − 4.4 and − 7.8 ϕ. Fragmented masses are similar across low intensity explosions at < 1 × 10^3^ kg. The number of particles fragmented from the rising magma mass does not exceed 400 at any recorded stage of these explosions, suggesting steady conditions.

All explosions are bomb-dominated. The greater spatial resolution for images recording low intensity explosions shows fragmentation only down to particle sizes of − 2 ϕ (< 10% of total number of particles in any image of these sequences), however the cumulative total mass of all particles − 5 ϕ and below account for < 2% of the erupted mass when recalculating distributions with the inclusion of tephra sampling data suggesting that GSD assessment is satisfactorily accurate. With high intensity explosions, a tailing off in the number of particles begins at − 4 ϕ, which represents the smallest resolvable bin. In considering mass, a similarity of < 2% of the erupted mass is again contained within bins of − 5 ϕ and finer when recalculating distributions with the inclusion of tephra sampling data. This suggests that although fine lapilli and ash sizes are smaller than the resolution of the camera, and the fine-grained tail of the distribution is not fully characterizable; the mass of these particles is insignificant to the total erupted mass, even.

**Figure 5:**
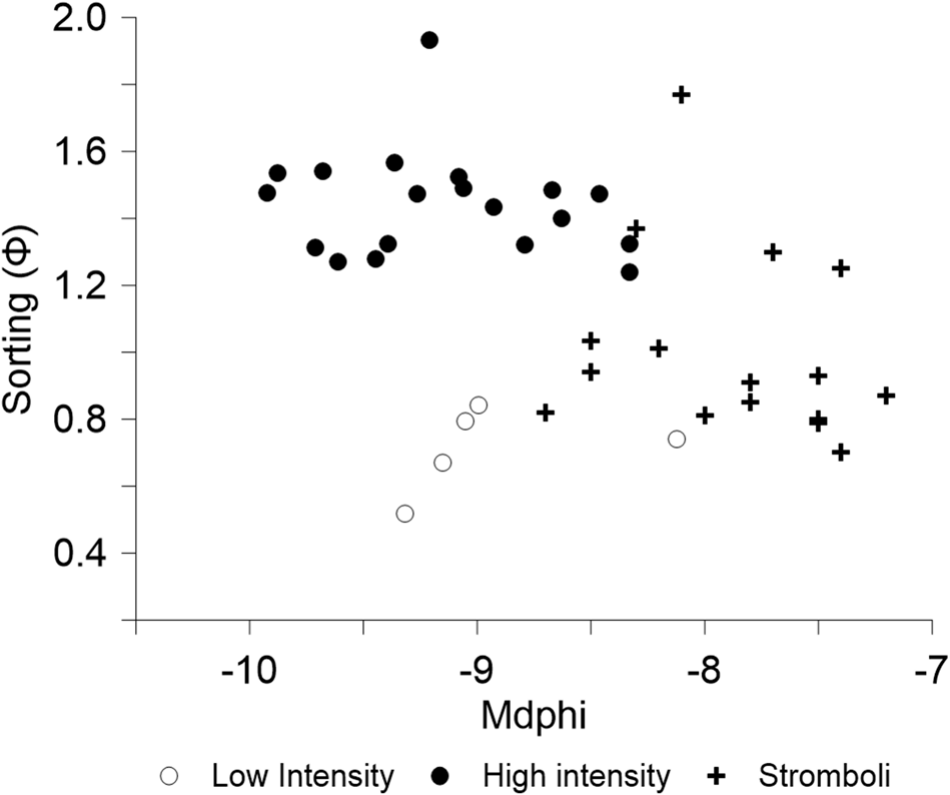
Particle size distributions by mass characterised by median phi and sorting for PdF explosions during the July–August 2015 eruption compared with the distributions of Stromboli 2012 explosions after^43^. The uncertainty in median particle size and sorting is smaller than the symbol size.

In all explosion sequences, GSDs tends towards finer particle size distributions over time. However, a coarsening of both particle median and maximum particle size is occasionally recorded by subsequent images of a sequence. When plotted in the Mdphi (Median of the mass distribution)-sorting (given by the difference between the 84th and 16th percentiles of the mass distribution divided by two^50,51^), the data clusters based on the two already identified explosion types (Fig. 5), suggesting that the two selected explosions categories are significant.

### Fragmentation dynamics

**Figure 6:**
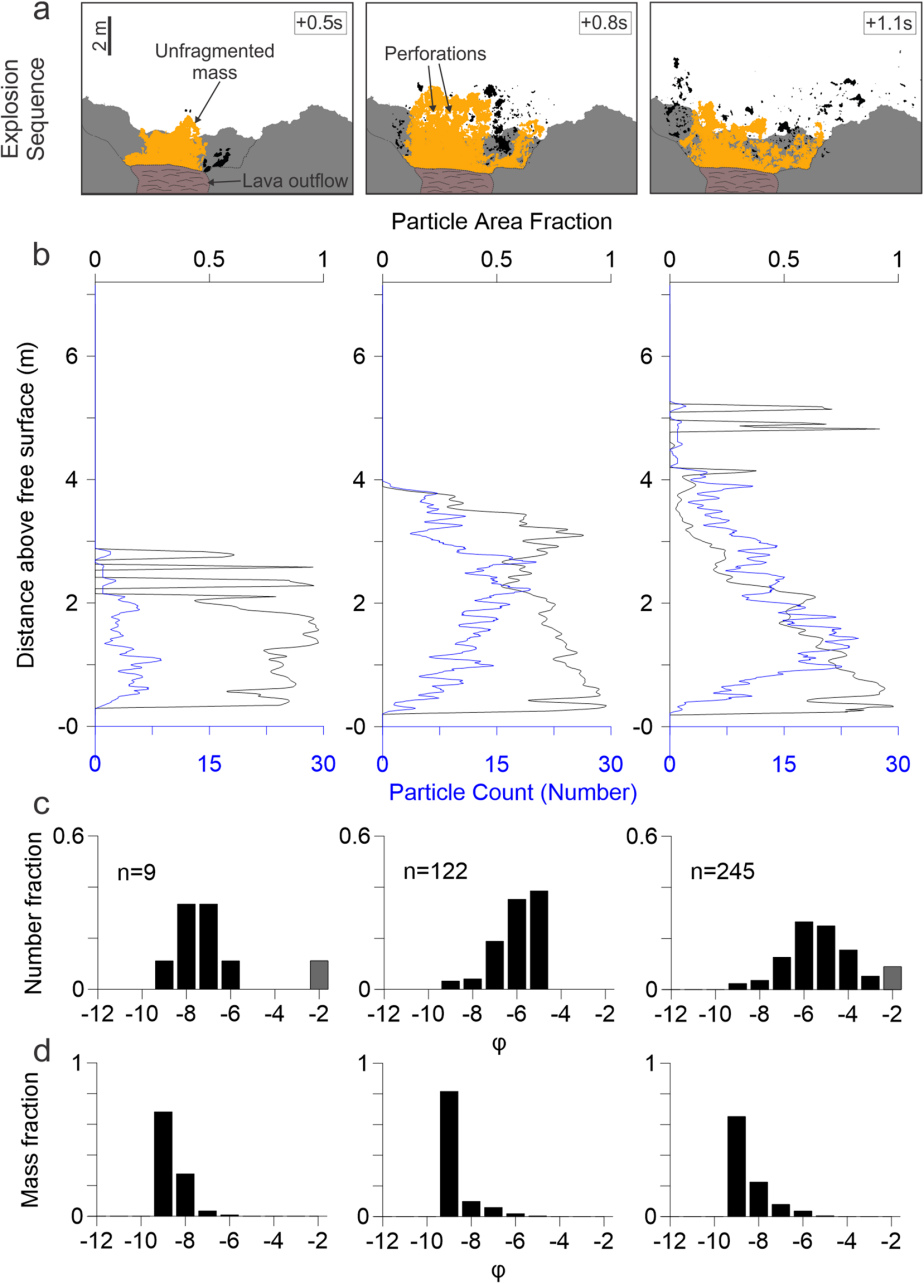
Explosion 2 sequence (**a**) with particles (black) and unfragmented mass (orange). Change in particle area fraction and particle count with height (**b**) and PSDs by number (**c**) and mass (**d**) over time. Grey bars indicate the frequency of sizes within the particle detection limit.

**Figure 7:**
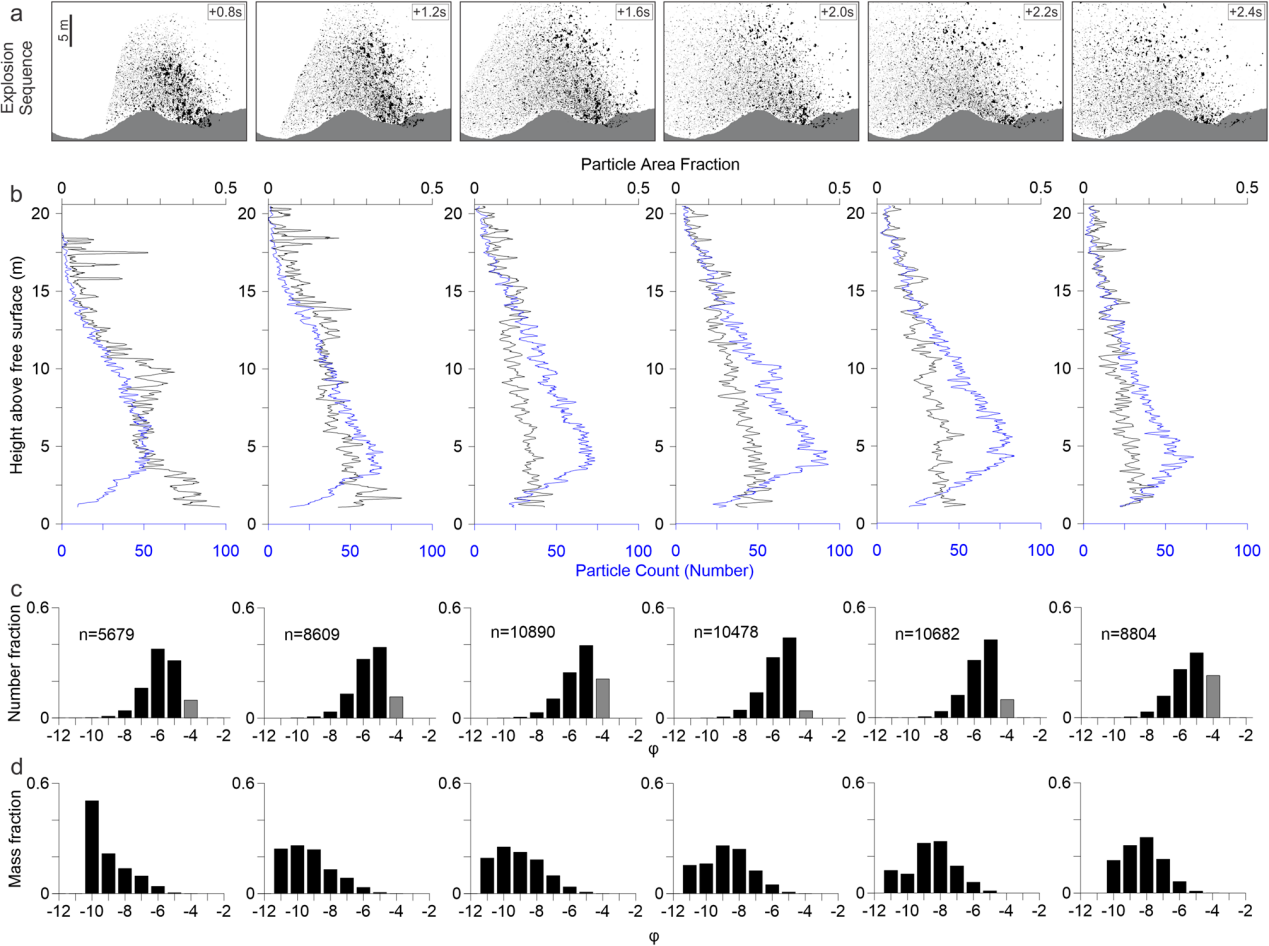
Explosion 6 sequence (**a**) with particles (black). Particle area fraction and particle count (**b**) with height and PSDs by number (**c**) and mass (**d**) over the sequence. Grey bars indicate frequency of sizes within the particle detection limit.

The following describes two explosion sequences, with the most complete image sequence for a low and a high intensity explosion, labelled explosion 2 and 6 respectively (Figs. 6 and 7 and Table 1).

**Figure 8:**
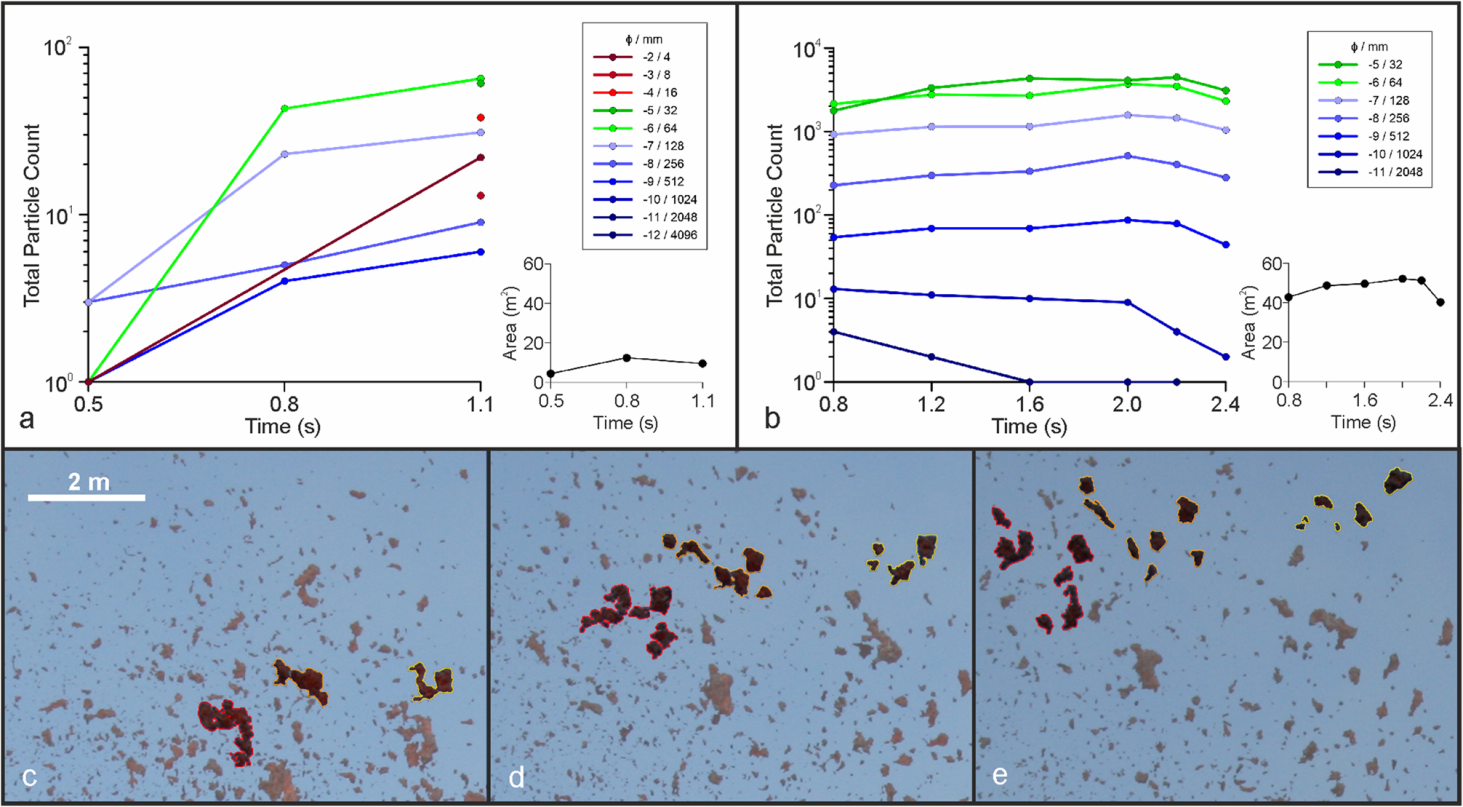
Evolution of particle counts by grain size for (**a**) low intensity and (**b**) high intensity explosions with inset plots of total area of fragments throughout each sequence. Secondary fragmentation within a high intensity explosion of three bombs splitting into ~ 6 particles each (**c**–**e**).

In explosion 2, the particle area fraction decreases over time (Fig. 6). The sequence begins with a poorly fragmented jet; a low number of particles is visible at the top and lateral margins of the jet (Fig. 6a). Consequently, the particle area fraction is uniformly high in the jet. After 0.8 s, the jet begins to break up predominantly along the jet margins, forming cm- to dm-sized fragments at both sides. A fracture across the leading edge begins to form at about 2 m above the vent but fails to develop into individual clasts. After 1.1 s, fragmentation is more pervasive resulting in a further increase in particle number and decrease in the particle area fraction as the jet expands. Through time, the number PSD shifts to larger ϕ (i.e. smaller particle sizes) than at the onset (Fig. 6b). The median particle size by number decreases from − 7.8 ϕ to − 6.3 ϕ between the first and second images, and is associated with an asymmetry in the shape of the distribution by number of particles (Fig. 6c). A more symmetrical distribution is reached by the final image, and median particle size by number shifts to − 5.8 ϕ. An increase in the − 2 ϕ class is observed in this image, however, due to the limits of image resolution, this bin may be artificially inflated by counts of sub-resolution particles. Particle counts for all ϕ classes generally increase over time (Fig. 8).

Mass PSDs are consistent over the sequence at − 9.2 to − 9.4 ϕ (Fig. 6d). The − 9 ϕ class consistently accounts for > 60% of the fragmented mass throughout the sequence. Particles fragmented represent only < 20% of the total rising mass, which is mostly erupted as a continuous jet falling back into the vent.

In explosion 6, the jet breaks up just above the free magma surface (Fig. 7a). The fragmented jet then poorly expands, resulting in relatively consistent particle area fractions across the sequence (Fig. 7). In parallel, the particle number also decreases linearly with height by 5 particles/m (Fig. 7b). With time the jet stabilizes into a constant expansion rate marked by a linear decrease of the particle density with height of 0.01/m. Median ϕ in number PSDs is similar in all images at − 5.7 to − 6.2 ϕ with sorting of 1.0 to 1.4 ϕ (Fig. 7c, Table 1). Two trends towards larger ϕ are present within the sequence.

Variations to particle counts per ϕ class are present across the image sequence (Fig. 8). Total counts of the smallest ϕ classes (largest particles) decrease while counts for the largest ϕ classes (smallest particles) increase over time. A decrease in all counts of the final image is representative of loss of particles through settling and/or their exceeding the field of view. In all classes, except − 4 ϕ, the particle count per ϕ class is greater than the next coarser class which is again a result of the image resolution limit (Fig. 8). As a consequence, the mass PSD is initially asymmetrical and gradually shifts over symmetrical shapes centered around − 9 ϕ (Fig. 7d). In the mass distribution, the Mdϕ varies from − 7.7 to − 8.3 ϕ. Mass PSDs are more variable than the low intensity explosions, shifting from − 10 to − 8.8 ϕ over the sequence. This corresponds to an initially asymmetrical distribution and gradually shifts over symmetrical shapes centered around − 9 ϕ. Particles fragmented represent > 95% of the total mass visible above the cone rim.

It is important to note that an unknown number of particles are lost from the field of view, primarily along the left and upper edges. We estimate these particles to be limited in number due to the approaching zero particle area density measured at the top of the frame (Fig. 7b). Particles lost to the left edge were dispersed by wind and therefore assumed to be predominantly composed of the finer ϕ classes; sheet sampling confirmed a median ϕ of − 4.0 and an absence of particles larger than − 5 ϕ at distances outside of the image field of view. Likewise these are not considered to be significant as to alter the mass distributions due to the small mass fractions associated with the − 4 and − 5 ϕ classes (Fig. 7d).

### Particle velocities

Exit velocities of magma in low intensity explosions range from 8.3 to 9.3 m/s based on extrapolated maximum heights of particles and unfragmented lava masses. Particle velocities in high intensity explosions suggest that the ejection of magma occurs over a timeframe of at least 0.6 s. The majority of measured particles have vent exit velocities in the range of 15–20 m/s and were erupted over a ~ 0.25 s window (0.57–0.83 s prior to the first image of the sequence). As already noted in other small-scale explosions^52–54^, exit velocities are the highest at the explosion onset (20–22 m/s), and decrease with time down to 6.5 m/s.

## Discussion

The July–August 2015 eruption of PdF was a type case of for the lowest-intensity end member of mafic explosions, where most of the magma was primarily erupted as lava flows and extremely low-energy explosivity was observed at the vents. These explosions are generally overlooked and poorly documented because they rarely form a definable tephra deposit that can be linked to a single event, with most scoria either falling and coalescing into lava flows or welding at the vent^55^, or being mixed for multiple (hundreds to thousands) of events. When deposits are formed, later activity often covers them. Thus, these very small-scale explosions can only be documented by observations and data collected during each individual event, such as occurred in this PdF eruption.

Images taken during the PdF eruption, when discrete explosions were safely observable at short distances from the vents provide for unique insight into determining the evolution of particle size distributions during magma fragmentation. For the lowest intensity explosions, only primary fragmentation (i.e. occurring from the magma jet) is captured in the images (Fig. 6). These likely represent the lowest intensity end-member of explosivity in a basaltic system, and are marked by incomplete magma fragmentation of small magma bursts fed by very low gas overpressure^30,56^. Particles fragmented from the periphery of the jet and perforations developed within the ascending magma. The magma failed to completely fragment and predominantly fell back into the vent. The fragmentation of a magma sheet was dominated by the combined effects of surface tension and magma stretching. Perforations (or Taylor rims) can form as the sheet expands^57^. As these rims grow, they connect, thereby fragmenting the sheet. In the ascending magma, perforations are observable towards the vertical edge (Fig. 6a). Here, however, there were insufficient time for the growth of the rims, so perforations appeared in the ascending magma, but the magma collapsed before the perforations could coalescence and cause significant fragmentation. The geometry and occurrence of these perforations suggest that they are linked to the Rayleigh-Plateau instability, which is dominated by capillary (surface tension) forces^58^.

The number of pyroclasts produced in any of the studied low intensity explosion images was limited to < 250 (and < 400 in the second analysed sequence), yet spanned a wide size range of − 2.8 to − 9.9 ϕ (Table 1). Size distributions by mass are dominated by the few fragments formed by primary fragmentation of the jet body, but number distributions are dominated by the (smaller) fragments formed by primary fragmentation of the magma at the jet peripheries^30,40^.

In contrast, the high intensity explosions evidence a more complete fragmentation. The largest bombs are of the order of the jet diameter in the first image (− 11 ϕ) and are therefore attributed to the primary fragmentation of the jet. The total number of pyroclasts produced was typically between one and two orders of magnitude greater than the low intensity particle counts, and are associated with a matched increase in the respective total fragmented masses (Table 1).

Fragmentation efficiency is usually correlated with the size of generated particles or the numbers of particles formed^59^, and increases with the amount of energy spent in the fragmentation process,in this work we define efficiency as the number of particles generated per m^3^ of magma, and derive this parameter directly from image analyses (Table 1). In the explosions analysed in this work, Mdϕ shows a limited variability independent of explosion intensity, being comprised between − 8.3 and − 10.1 ϕ (i.e. 32–110 cm). A wider range of median phi of − 4.4 to − 7.8 ϕ (i.e. 2–22 cm) for number distribution is present, with the smallest median size of − 4.4 ϕ associated with the incomplete magma fragmentation of a low intensity explosion—large particles were observed to be forming but failed to fragment completely from the rising mass (Fig. 6a). However, the typically incomplete fragmentation of the mass in low intensity explosions means the fragmented particles account for a maximum of only < 20% of the total mass. That is, fragmentation does not proceed to a low particle (ash) particle size, as has also been observed for jets at Stromboli^40^. All other explosions have Mdϕ larger than − 5.4 ϕ.

Median particle sizes by mass and number overall shifted towards finer phi as explosion sequences progress and fragmentation continued (Table 1). Occasional coarsening of median phi represents fragmentation of the previously unfragmented magma mass in the interim of the two images. The overall trend is similar regardless of eruption intensity, with median phi of the low intensity explosions in the final sequence image nearing or matching that of high intensity explosions. This suggests that despite the difference in ‘completeness’ of fragmentation (i.e. the proportion of magma that fragmented into discrete particles versus that which remained connected to the free surface and fell back into the vent, Table 1), the fragmentation efficiency is comparable regardless of the explosion intensity. However, this contrasts with the expectation that higher intensity explosions are generated by higher energies and thus should result in finer particle size distributions. We suggest that the increase in mass ejected in the high intensity eruptions accounts for the similarity in fragmentation efficiency as the increased mass requires increased energy to fragment it to the same size distributions observed in the low intensity eruptions. This is an important point that also should make us reflect on how we define the fragmentation level in such low explosivity activity. If the fragmentation level is the boundary marking the passage from a continuous liquid with dispersed bubbles to fragments of magma within gas, but fragmentation occurred gradually across a discrete zone, we should rather use the concept of *fragmentation region* where the phenomenon progresses to its full extent. In that case, as was also proved for Strombolian explosions at Stromboli, where for the lowest intensity explosions, fragmentation energy does not scale with the mass and the particle size, but to the gas energy^60,61^. Individual explosions of high intensity are relatively similar in terms of grainsize but vary up to two order of magnitude in the total fragmented mass.

When early stages of the explosion were captured and fragmentation is incomplete, particle size distributions show asymmetrical shapes with fine-grained tails (Fig. 7d). We associated these with predominantly primary magma fragmentation. As explosions progressed, the shape of the distributions shift towards more symmetrical distributions (Fig. 7d). We associate these with predominantly secondary magma fragmentation (i.e. breakup of already fragmented clasts), which is distinct from brittle fragmentation of clasts at the impact with the ground^55^.

The evolution of PSDs by primary and secondary fragmentation is visible by comparing particle counts within phi classes over time (Fig. 8). In the low intensity sequence, all phi classes initially increase as particles fragment into scoria at the peripheries of the magma (Fig. 6a and 8a). These continue to increase with further fragmentation of the mass and only minor secondary fragmentation of particles visible.

This secondary fragmentation is more pronounced in the high intensity sequence (Fig. 8b). The largest size classes (− 11 and − 10 phi) progressively decrease in particle counts over time, while being associated with an increase in particle counts of all other classes. After 1.4 s all particle counts per class with the exception of − 5 phi begin to decrease, primarily due to settling of the particle cloud from this point. All classes have decreased in the final captured image of the sequence.

The relatively stable mass calculated for each image prior to settling, alongside the count variation per class indicates that fragmentation is occurring in the absence of the addition of new mass. This secondary fragmentation is directly observable in bombs across successive images of the sequence. Large particles fragment to multiple smaller particles. Newly formed lapilli are roughly equidimensional (Fig. 8c–e).

With this study we can thus provide data not only on the median grainsize of the particles, but we can also quantify the size distribution and how it changes with time during the explosion and height above the vent. Although analysis of images of volcanic eruptions is common, grain size distribution data is not regularly obtained this way and subsequently published. It is therefore not possible to directly compare the PdF 2015 GSDs with similar scale explosions. Particle data determined for higher intensity Stromboli explosions via thermal image analysis ^34^ and now computed in terms of Mdϕ and sorting and are shown alongside the PdF data, displaying a finer median particle size than determined for the PdF explosions but spanning the same range of sorting (Fig. 5). The higher fragmentation efficiency could be derived from more significant gas shearing effects on the magma surface^35^.

GSDs of larger mafic explosions have been determined based on the associated deposits^7, 10,62^, and are not directly comparable with the PdF results. They show, however, typically finer Mdϕ (− 4.1 ϕ for the 1986 Izu Oshima eruption to 0.7 ϕ for the 2006 Etna eruption) and very variable sorting^63,64^. Based on the consideration above, it is difficult to interpret this variability directly in terms of fragmentation dynamics and/or eruptive conditions.

Because of the limited number of explosions studied here and of published studies on other events, it is not possible to generalise our results to derive a model on fragmentation dynamics. Viscosities of the Piton magmas are too low to allow for brittle fragmentation^22^. A combination of low Reynolds number and Oh on the order of 10^0^–10^1^ suggests that surface tension processes still play a fundamental role in the fragmentation mechanism^26,27^ but inertial forces ultimately drive the breakup of the jet^28^. For smaller Oh (i.e. lower viscosities) magma fragmentation processes would be dominated only by surface tension forces^25^ with formation of particles with shapes suggesting ligament mediated fragmentation such as Pele’s hair and tears^24^ which characterise even more energetic fountains^65^ but were not retrieved in this eruption.

## Conclusions

This study details the lowest-intensity type of basaltic explosive eruption, an end-member which is extremely poorly defined. These are the lowest energy end-member of mafic explosive eruptions, and are marked by intermittent, but extremely frequent explosions with coeval lava flows. Events disperse material only a few tens of meters from the vent and, because of the low density of the associated fountains and the small number of particles formed, constitute an ideal case for analysing the dynamics of low viscosity (10^1^–10^2^ Pa s) magma fragmentation. We thus investigated the importance of primary and secondary fragmentation on the size distribution of particles formed during these exceedingly common types of explosions, where events (locally and globally) proceed at a rate of thousands per hour^66^.

Based on the results obtained on a representative dataset of selected explosions which occurred at the fissure eruption of Piton de la Fournaise volcano in July–August 2015, we can conclude that:

- Magma fragmentation can vary from incomplete (it does not involve the entire ejected mass) in the lowest intensity explosions to complete in the highest intensity explosions. This ‘completeness’ is not associated with variability of the fragmentation efficiency, which was highly similar, resulting in comparable median diameters of the particles generated by the explosions, regardless of explosion intensity.
- Particle size distributions become progressively more influenced by secondary fragmentation with increasing jet/particle velocities. Short-timescale low intensity explosions where rising magma has insufficient time to fragment before collapsing produced PSDs dominated by primary fragmentation. In longer lasting high intensity explosions where particles are ejected tens of metres high, secondary fragmentation shifts PSDs to finer sizes.

Data for a greater variety of events are necessary to understand how fragmentation could be affected by variations in magma viscosity due to crystal and bubble content variations within the basaltic magma type considered here. However, we show that accurate and useful surveillance products can be obtained by application of appropriate processing to eruption monitoring data (e.g., visible and thermal video), without a need for recourse to deposit-based studies. Such output can be used to allow this low intensity, but exceedingly common, style of explosive activity to be tracked and monitored in real-time. Finally, we provide a detailed description of the fountain dynamics coupled with quantification of the main dimensionless parameters able to characterise fragmentation dynamics. These data were useful to define the role of the main forces controlling clast formation within these low energy fountains.

## Methods

### Field campaign/image recording

A field campaign conducted on July 31-August 3 2015 recorded images and videos of explosions and lava flows from three vents between 12:18 pm and 13:13 pm of August 1 (local time). Two cameras were used in this study at locations marked in Fig. 1c with positions determined by GPS with 1 m accuracy (Table 2). The first was a handheld Canon EOS Digital Rebel XTi (with a Canon EF-S 18–55 mm f/3.5–5.6 IS II lens) which acquired 3,888 × 2,592 pixel jpeg images. 72, 12 and 69 images were taken of explosions occurring at Cones A, B-I and B-II respectively (Fig. 1). The second was a forward looking infrared (FLIR) camera—model T650sc, which acquired 640 × 480 pixel images at wavelengths of 7.5–13 µm. A total of 124 images of explosions, 307 images of the lava flow from Cone A and two lava flow videos were taken with this camera. Measurements of lava temperature, lava flow velocity and lava channel dimensions were obtained from the FLIR camera images^67^. Temperatures were corrected for emissivity and atmospheric effects using the FLIR systems on-board correction facility setting an emissivity for basalt of 0.98^68^. Image scales were calculated from known distances between vents and cameras, and image metadata including the focal lengths, camera optics and camera sensor sizes. Pixel sizes in images ranged from 4.9 to 12.2 ± 0.8 mm depending on the camera location and the vent being recorded. Full details of pixel size and equivalent field of view are given in Table 2.

**Table 2:** Camera locations and properties of images taken for given camera-distance relationships.

Camera	Position (UTM coordinates)		Distance [to cone] (m)	Pixel size (mm)	Focal plane field of view (m)
	**E**	**S**			
FLIR	366,918.66	7,652,928.06	[A] 239.8	169.5 ± 5.5	108.5 ± 3.6 × 81.3 ± 2.5
	366,788.00	7,652,779.39	[A] 47.5	33.6 ± 5.5	21.5 ± 3.6 × 16.1 ± 2.5
Canon	366,788.00	7,652,779.39	[A] 47.5	4.9 ± 0.8	19.2 ± 3.2 × 12.8 ± 2.2
	366,610.67	7,652,511.85	[B1] 115.8	12.0 ± 0.8	46.7 ± 3.2 × 31.2 ± 2.2
			[B2] 117.5	12.2 ± 0.8	47.4 ± 3.2 × 31.6 ± 2.2

Several water-quenched bombs were collected from spattering at Cone A and B-II; these were analysed for density, crystallinity, vesicularity and composition (bulk, crystal and groundmass) and grain size distribution in previous researches; with results published and freely accessible in the open database DYNVOLC^35,45^. Scoria lapilli were sampled on July 30, August 1, and 3 on a 20 by 20 cm wide plastic sheet located at 50–100 m from Cones A and B-I^45^. The physical parameters measured from the pyroclasts emitted the second day from the very mild explosions, were used to estimate the fluid viscosity^69^. We use the Einstein-Roscoe relationship to estimate the effect of crystallinity, account for the effect of sheared and unsheared bubbles^47^, and for the three phase mixtures^48^ as tested on, and applied to, active lavas^70^ and on pyroclasts^71^. Lapilli shapes were quantified by measuring three perpendicular axes of selected clasts which did not show broken faces.

Reynolds (Re) and Ohnesorge (Oh) numbers were calculated as:

$$Re = \frac{{{\rho _m}UD}}{\eta }$$

and

$$Oh=\frac{\eta }{\sqrt{{\rho}_{m}\sigma D}}$$

With *ρ*_*m*_ = magma density (1,320 ± 40 kg/m^3^), *η* = magma viscosity (27–370 Pa s), *U* = exit velocity (8.3–21.2 m/s), *σ* = surface tension (0.08 N/m), *D* = jet diameter (1.0 ± 0.2 m). These parameters are expected to have changed within the jet over time and with height through cooling, deceleration and jet breakup. Our values represent the magma parameters at the vent exit, and therefore are at a maximum.

### Image selection, preparation and processing

Data on eruption parameters was sourced primarily from thermal camera imagery which imaged the magma within the vent of Cone A and contemporaneous lava flow flowing through the NW crater rim of the same vent.

Images of explosions were selected for analysis in order to obtain the most accurate dataset as possible. In total we chose 21 images across six explosions image sequences, and an additional three images of three other explosions, avoiding all images showing a significant number of particles from previous or subsequent explosions (i.e. non-homogenous particle areal distributions suggesting multiple pulses) and obtaining full temporal coverage of discrete explosions.

Images from each sequence were first aligned using image processing software before being cropped to ensure each frame was contained within the aligned sequence. For Cone B-I and B-II a pseudo-background was created for each image in ImageJ by creating duplicate images with a Gaussian blur of 50. This was necessary due to the blue sky gradient in each image and subsequent difficulty in isolating particles by simple thresholding. The cropped image was then subtracted from the blurred duplicate and inverted to display the particles and the land mass against a pure white background. The land mass was manually removed and particles below the horizon were manually selected and added to the particle image. Particles which appeared to originate from earlier explosions were also removed (i.e. clustering separately from the main fountain structure). The image was finally converted to a binary image for particle analysis in MATLAB.

For Cone A, particles were isolated directly using colour thresholding, exploiting the red colour of uncooled fragmented magma from the black-brown cooled particles of previous explosions and cone background.

As a proxy for the volumetric density of particles in the fountain, particle area fraction in each binary image was determined by calculating the ratio of black (particle) to white (non-particle) pixels in each row. The particle plume was considered to be bounded by the left-most and right-most particle per row and therefore white pixels on the left and right of these particles were not considered in calculations. Particle counts were calculated per row by totalling the number of times sequential black pixels were present in each row. The land mass was excluded from the particle counts when particles were lower than the cone rim. A moving average of 10 was applied to both the particle area fraction and particle count output.

Measurements of particle areas, Feret diameters and minimum Feret diameters were performed in ImageJ for every particle within each binary image. Based on shape analysis made on the scoria collected on July 31, August 1 and 3 (Fig. 4), particle volume was calculated by multiplying the particle area by the minimum Feret diameter^72^ and converted to a mass given the scoria density. Given the density distribution available and the size range of the particles considered, which is much larger than the size of the vesicles in the scoria^34^, the density variations are neglected. The full grain size distribution data for explosions 2 and 6 are provided as supplementary material.

The unfragmented mass (where no separation between the ejected magma and the rim of the cone was present) was excluded from particle analysis but accounted for in calculating the total erupted mass. Selected particles were manually tracked using the ImageJ plugin MTrackJ^73^ to provide above vent heights and particle velocities. Particle exit velocities were derived by calculating their reverse path given instantaneous velocities and positions (above the vent) of particles and considering gravitational effects.

Uncertainty on size distribution was compared with distributions of the clasts collected close to the vent^45^ (Fig. 2). These PSDs are considered as complementary to our image analyses, as the deposits lack the largest clast which fell back on the vent and were incorporated in lava flows, and comprise the fraction of particles which are smaller than the pixel size. For each image analysed, virtual PSDs were constructed by artificially adding a fine tail with the shape of the PSDs of the deposit. In terms of mass these distributions differ of up to 1.4%, and Mdϕ and sorting differed only of up to 0.1 ϕ with respect to the PSDs obtained by image analysis. In terms of number of particles, the difference is much more important: the median size coincides with the smallest size retrieved at the deposit (2–3 ϕ) and the distribution become bimodal with the main peak at these same sizes. For these reasons, mass PSDs will be considered for comparison with other deposits, while number PSDs will be used only to describe fragmentation dynamics and for inter-eruption comparisons.

## Supplementary information

Supplementary information.
