# Supplementary material for: Magma fragmentation and particle size distributions in low intensity mafic explosions: the July/August 2015 Piton de la Fournaise eruption
Source: Sci Rep. 2020 Aug 18;10:13953. doi: 10.1038/s41598-020-69976-y (PMC7434896; doi:10.1038/s41598-020-69976-y)
Supplement: Supplementary file 1 — Supplementary information. [file 41598_2020_69976_MOESM1_ESM.pdf]

Magma fragmentation and particle size distributions in low intensity mafic explosions: the July/August 2015 Piton de la Fournaise eruption

Matthew J. Edwards, Laura Pioli, Andrew J.L. Harris, Lucia Gurioli, Simon Thivet

SUPPLEMETARY TABLES AND FIGURES

Cone	Time (GMT 08 Aug 2015)	Explosion number	Image in sequence	Mass Percentage (Phi)										
				-12	-11	-10	-9	-8	-7	-6	-5	-4	-3	-2
A	8:19:26	1	1	0.0	0.0	0.0	0.0	70.9	18.5	7.7	2.6	0.2	0.0	0.0
	8:19:27		2	0.0	0.0	0.0	61.5	23.8	10.2	3.4	0.9	0.1	0.0	0.0
	8:19:28	2	1	0.0	0.0	0.0	68.1	27.6	3.5	0.9	0.0	0.0	0.0	0.0
	8:19:29		2	0.0	0.0	0.0	81.7	9.9	6.0	1.9	0.5	0.0	0.0	0.0
	8:19:30		3	0.0	0.0	0.0	65.2	22.5	8.0	3.6	0.6	0.0	0.0	0.0
B-II	8:55:16	3	1	0.0	0.0	0.0	34.0	28.3	22.6	12.5	2.5	0.1	0.0	0.0
	8:55:17		2	0.0	0.0	29.6	26.3	19.9	16.0	7.0	1.2	0.1	0.0	0.0
	8:55:18		3	0.0	0.0	16.2	25.4	25.5	21.7	9.7	1.4	0.1	0.0	0.0
	9:00:14	4	1	0.0	11.0	18.8	31.6	24.8	11.1	2.5	0.3	0.0	0.0	0.0
	9:00:15		2	0.0	22.9	30.5	20.1	16.1	8.0	2.1	0.3	0.0	0.0	0.0
	9:00:16		3	0.0	11.7	38.0	21.3	17.5	8.5	2.5	0.4	0.0	0.0	0.0
	9:05:05	5	1	0.0	0.0	16.7	31.3	21.2	15.9	10.7	4.0	0.2	0.0	0.0
	9:05:05		2	0.0	0.0	8.2	22.1	30.3	23.4	12.2	3.6	0.2	0.0	0.0
	9:05:06		3	0.0	0.0	15.2	19.4	26.6	23.0	12.4	3.1	0.2	0.0	0.0
	9:06:51	6	1	0.0	0.0	50.6	21.7	13.7	9.6	3.9	0.5	0.0	0.0	0.0
	9:06:51		2	0.0	24.3	26.1	24.0	13.1	8.5	3.4	0.6	0.0	0.0	0.0
	9:06:51		3	0.0	19.4	25.3	22.5	18.5	9.7	3.6	0.9	0.0	0.0	0.0
	9:06:52		4	0.0	15.4	16.2	26.1	24.2	12.4	4.8	0.8	0.0	0.0	0.0
	9:06:52		5	0.0	12.4	10.4	27.2	28.2	14.8	5.9	1.2	0.0	0.0	0.0
	9:06:52		6	0.0	0.0	17.9	25.9	30.3	18.5	6.2	1.1	0.1	0.0	0.0
	9:06:35	7	-	0.0	0.0	38.2	27.6	15.1	8.5	8.6	2.0	0.0	0.0	0.0
	9:06:30	8	-	0.0	19.7	14.2	18.2	21.1	15.9	8.2	2.5	0.2	0.0	0.0
	9:11:48	9	-	0.0	0.0	24.4	24.8	25.2	18.3	6.3	0.9	0.1	0.0	0.0

Table 1. Full GSD data by particle mass.

Cone	Time (GMT 08 Aug 2015)	Explosion number	Image in sequence	Number Percentage (Phi)										
				**-12**	**-11**	**-10**	**-9**	**-8**	**-7**	**-6**	**-5**	**-4**	**-3**	**-2**
A	8:19:26	1	1	0.4	0.0	0.0	0.0	4.0	8.1	18.4	33.2	17.5	9.0	9.4
	8:19:27		2	0.3	0.0	0.0	1.8	4.3	9.1	18.5	28.6	22.0	6.8	8.6
	8:19:28	2	1	0.0	0.0	0.0	11.1	33.3	33.3	11.1	0.0	0.0	0.0	11.1
	8:19:29		2	0.0	0.0	0.0	3.3	4.1	18.9	35.2	38.5	0.0	0.0	0.0
	8:19:30		3	0.0	0.0	0.0	2.4	3.7	12.7	26.5	24.9	15.5	5.3	9.0
B-II	8:55:16	3	1	0.0	0.0	0.0	0.4	2.5	10.7	33.9	40.2	12.3	0.0	0.0
	8:55:17		2	0.0	0.0	0.1	0.6	2.7	12.3	34.0	38.0	12.4	0.0	0.0
	8:55:18		3	0.0	0.0	0.0	0.3	2.5	12.2	33.7	34.3	17.0	0.0	0.0
	9:00:14	4	1	0.0	0.0	0.2	1.2	6.7	20.4	30.6	23.8	17.1	0.0	0.0
	9:00:15		2	0.0	0.0	0.2	1.1	5.5	17.3	30.8	28.3	16.7	0.0	0.0
	9:00:16		3	0.0	0.0	0.2	0.9	4.2	13.0	27.4	31.1	23.1	0.0	0.0
	9:05:05	5	1	0.0	0.0	0.0	0.2	0.9	5.2	20.3	46.1	27.3	0.0	0.0
	9:05:05		2	0.0	0.0	0.0	0.2	1.2	6.8	22.9	43.1	25.8	0.0	0.0
	9:05:06		3	0.0	0.0	0.0	0.1	1.2	6.4	22.0	39.3	30.9	0.0	0.0
	9:06:51	6	1	0.0	0.1	0.2	1.0	4.0	16.2	37.6	31.2	9.7	0.0	0.0
	9:06:51		2	0.0	0.0	0.1	0.8	3.5	13.2	32.1	38.6	11.6	0.0	0.0
	9:06:51		3	0.0	0.0	0.1	0.6	3.1	10.5	24.8	39.6	21.3	0.0	0.0
	9:06:52		4	0.0	0.0	0.1	0.7	4.4	14.0	33.0	43.8	4.0	0.0	0.0
	9:06:52		5	0.0	0.0	0.0	0.6	3.5	12.3	31.3	42.4	9.9	0.0	0.0
	9:06:52		6	0.0	0.0	0.0	0.5	3.2	11.8	26.3	35.3	22.9	0.0	0.0
	9:06:35	7	-	0.0	0.0	0.1	0.4	1.5	7.5	35.0	55.1	0.4	0.0	0.0
	9:06:30	8	-	0.0	0.0	0.0	0.2	1.2	5.7	20.0	39.3	33.6	0.0	0.0
	9:11:48	9	-	0.0	0.0	0.1	0.4	2.9	12.2	28.5	30.3	25.7	0.0	0.0

Table 2. Full GSD data by number of particles.

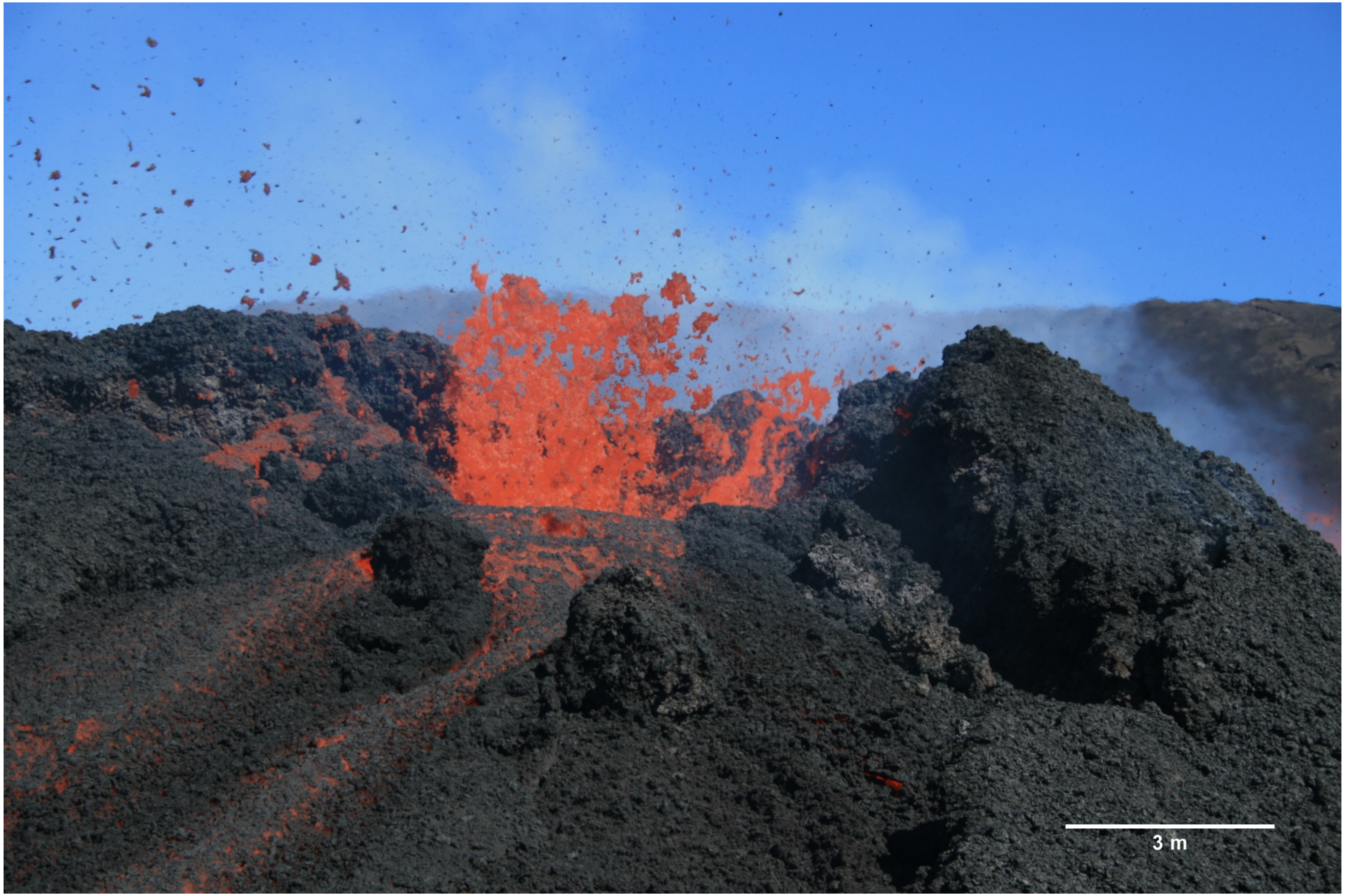

Figure 1. Representative image of a low intensity explosion at Cone A. Image displayed corresponds to Explosion 1, Image 2.

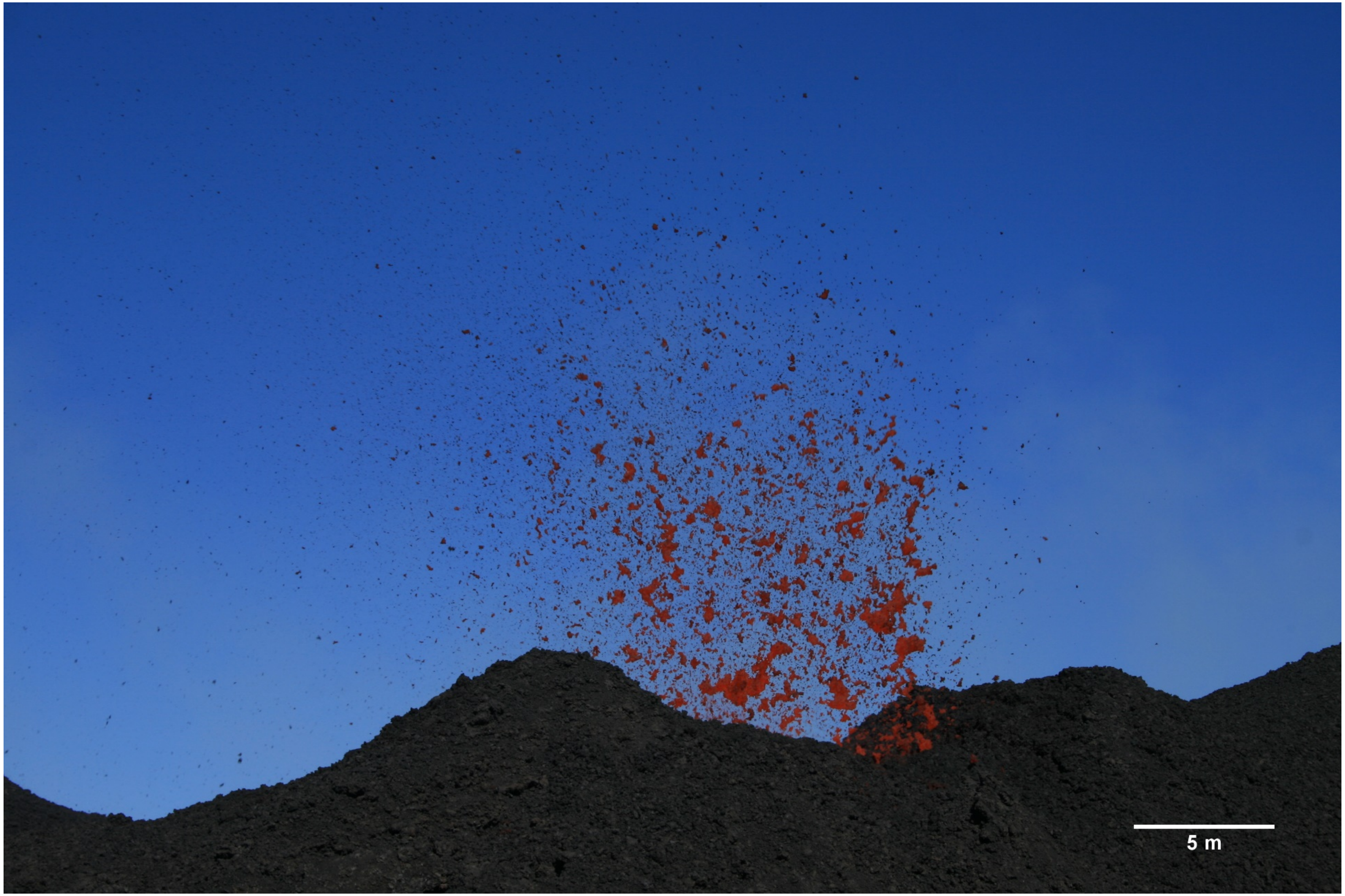

Figure 2. Representative image of a high intensity explosion at Cone B-II. Image displayed corresponds to Explosion 5, Image 1.
